# Origin of Large Second-Harmonic Generation in Nonpolar Molybdenum Tellurite Compounds

**DOI:** 10.3390/molecules31050787

**Published:** 2026-02-26

**Authors:** Zhian Li, Xiyue Cheng, Qian Xu, Xiu Wang, Guoliang Liu, Shuiquan Deng

**Affiliations:** 1 State Key Laboratory of Functional Crystals and Devices, Fujian Institute of Research on the Structure of Matter (FJIRSM), Chinese Academy of Sciences (CAS), Fuzhou 350108, China; lizhian@fjirsm.ac.cn (Z.L.); xuqian@fjirsm.ac.cn (Q.X.); wangxiu@fjirsm.ac.cn (X.W.); liuguoliang@fjirsm.ac.cn (G.L.); 2 College of Chemistry and Materials Science, Fujian Normal University, Fuzhou 350117, China; 3 Fujian College, University of Chinese Academy of Sciences, Beijing 100049, China; 4 College of Physics and Energy, Fujian Normal University, Fuzhou 350117, China

**Keywords:** second-harmonic generation, nonlinear optical material, density functional theory, atom response theory

## Abstract

Molybdenum tellurite compounds have attracted increasing interest as promising nonlinear optical (NLO) materials, yet their microscopic second-harmonic generation (SHG) mechanisms remain unclear. In this work, the electronic structures and SHG responses of ATeMoO_6_ (ATM, A = Mg, Cd, Zn) are systematically investigated using first-principles calculations combined with atom response theory. The results show that the SHG responses are mainly governed by the occupied nonbonding O 2p states and the unoccupied Mo 4d and Te 5p states. Our atom response theory analysis reveals that a strong synergistic effect between stereochemically active lone pairs (SCALPs) on Te atoms and nonbonding O 2p states critically enhances the SHG response in ZnTM and MgTM. In contrast, the relative weaker Te SCALPs in CdTM fail to provide a comparable contribution, leading to its lower SHG performance. The structure group analysis reveals that MoO_4_ units dominate the SHG response, while TeO_4_ units provide secondary contributions. Moreover, group dipole moments are found to be insufficient to explain the SHG behavior. These findings provide microscopic insights into SHG origins and offer guidance for NLO material design.

## 1. Introduction

Nonlinear optical (NLO) crystals are essential for frequency conversion in the mid-infrared (mid-IR) region, which is of great technological importance for applications such as spectroscopy, biomedical imaging, and environmental monitoring [1,2,3]. To date, only a few of mid-infrared NLO crystals have achieved commercial application. For instance, ZnGeP_2_ and AgGaS_2_, despite their large second-harmonic generation (SHG) coefficients, commonly suffer from inherent drawbacks such as low laser-induced damage thresholds or poor thermal stability. Moreover, the growth of large, high-quality single crystals continues to pose significant challenges in terms of scalability, yield control, defect regulation, and cost management, factors that severely challenge their industrial adoption [4,5,6,7,8,9,10,11,12]. As a result, numerous promising candidates remain confined to laboratory research due to persistent synthesis and processing difficulties [13,14]. Consequently, the discovery of new high-performance mid-IR NLO crystals that can meet the demands of modern industry remains a critical scientific challenge to be overcome.

In recent years, the exploration of NLO crystals has attracted considerable attention. Among these, quaternary molybdenum tellurite systems [15,16,17,18,19] have gradually emerged as a research hotspot due to their excellent SHG performance. These systems contain cations susceptible to second-order Jahn–Teller (SOJT) distortion [1,20,21], specifically octahedrally coordinated d^0^ transition metals (such as Nb^5+^, V^5+^, Mo^6+^, and W^6+^) and cations with stereoactive lone pairs (such as I^5+^, Te^4+^, Se^4+^, and Sb^3+^). Both types of cations typically reside in asymmetric coordination environments. In 2006, Eun et al. [22] reported the noncentrosymmetric monoclinic compound Na_2_Te_3_Mo_3_O_16_ (space group: *I*2), which exhibits an exceptionally large SHG response of approximately 500 × α-SiO_2_. Subsequently, Zhou et al. [23] reported Ag_2_Te_3_Mo_3_O_16_, which also crystallizes in the *I*2 space group and shows an SHG intensity of about 8 × KDP. In addition, Yu et al. [15] reported BaTeMo_2_O_9_ with the *C*2 space group, whose effective nonlinear coefficient reaches 10.3 pm·V^−1^. Feng et al. [17] synthesized Cs_2_TeMo_3_O_12_ single crystals belonging to the hexagonal *P*6_3_ space group, for which the NLO coefficients *d*_32_ and *d*_33_ were measured to be approximately 6.8 and 6.5 pm·V^−1^, respectively, indicating favorable nonlinear optical performance. Wu et al. [24] further reported novel mid-infrared NLO crystals Ca_3_(TeO_3_)_2_(MO_4_) (M = Mo, W). Both compounds crystallize in the polar monoclinic space group *P*2_1_, with SHG responses reaching 3.5 and 2.8 times that of KDP for the Mo- and W-based compounds, respectively, demonstrating promising potential for mid-infrared applications. Overall, the reported polar molybdenum tellurites generally exhibit strong SHG responses.

In particular, the nonpolar molybdenum tellurite family ATM (A = Mg, Cd, Zn; TM = TeMoO_6_) has emerged as a promising mid-IR NLO system, exhibiting unexpectedly large SHG responses. These materials provide an excellent platform for investigating the microscopic origin of SHG beyond conventional macroscopic polarity considerations [25,26,27,28,29]. As early as 1980, Forzatti et al. [30] were among the first to synthesize MgTM and related compounds using solid-state reactions and high-temperature solution (flux) methods. In 2012, Zhang et al. [25] reported the growth of MgTM single crystals with SHG responses up to 1.5 × KTP. Based on local dipole moment calculations, they suggested that the strong SHG response of MgTM mainly originates from three NLO-active structural units, namely TeO_4_, MoO_4_, and MgO_6_ polyhedra. Later, Zhao et al. [26] synthesized CdTM single crystals and reported a large SHG response (2 × KTP) and found that the TeO_4_ and MoO_4_ polyhedra contribute more significantly to the SHG response than the CdO_4_ unit. Subsequently, Li et al. [28] successfully grew large CdTM single crystals using an improved top-seeded solution growth method. The powder SHG intensity was reported to be approximately twice that of KTP, with a nonlinear optical coefficient *d*_36_ of 8.5 pm·V^−1^. Additionally, they also pointed out that the strong SHG response originates from the cooperative contributions of three asymmetric structural units. In the same period, Zhao et al. [27] reported the successful growth of ZnTM single crystals with an SHG efficiency of approximately 10.5 times that of KDP, which was associated with the high polarizability of three asymmetric structural units. Although their fundamental structural units have been highlighted in prior studies, the microscopic origin of the SHG effect in the ATM family, as well as the specific structural motifs that dominate the overall NLO response, remain unresolved.

Theoretically, Jiang et al. [31] investigated the NLO properties of these ATM compounds using systematic first-principles calculations combined with an atomic-cutting analysis. The calculated SHG coefficients *d*_14_ of MgTM, CdTM, and ZnTM are 14.04, 11.75, and 10.61 pm·V^−1^, respectively, showing that nonpolar molybdenum tellurite crystals can still exhibit strong SHG responses, following the trend MgTM > CdTM > ZnTM. They further suggested that the SHG intensity does not depend on the intrinsic static dipole moment of the crystal structure but mainly arises from the favorable molecular-level superposition of MoO_4_ tetrahedra. Moreover, Cammarata et al. [32] combined experimental measurement and first-principles calculations to elucidate the origin of the SHG response in nonpolar ATM compounds. Their calculations yielded effective nonlinear coefficients $d_{eff}^{p}$ of 9.61, 16.96, and 12.65 pm·V^−1^ for Mg, Cd, and Zn compounds, respectively, indicating the trend CdTM > ZnTM > MgTM. They further demonstrated that the SHG response is primarily determined by the degree of orbital hybridization in the A–O bonds, rather than by the intrinsic structural dipole moment or the magnitude of noncentrosymmetric distortion. Despite previous investigations, the physical origin of the NLO response in these crystals remains debated, and systematic theoretical studies elucidating the underlying structure–property correlations are lacking. Consequently, a detailed analysis of the SHG origin at atomic and orbital levels is essential to understand the observed NLO performance.

In this study, we employed first-principles calculations combined with partial response functional (PRF) and atom response theory (ART) [33,34] to investigate the electronic structures and the origin of the SHG response in the molybdenum tellurite series. Our results indicate that the MoO_4_ groups contribute substantially more to the SHG response of the ATM compounds than the TeO_4_ groups, suggesting that Mo-centered polyhedra are the primary structural units responsible for the strong nonlinear optical activity in these materials. The strength of stereochemically active lone pairs (SCALPs) on Te atoms is identified as an essential factor modulating the SHG response in these compounds as an effective and positive synergy with O 2p states amplifies the response in ZnTM/MgTM, whereas relative weak Te SCALPs in CdTM lead to a weaker effect. We further found that dipole moments alone are insufficient to account for the strong SHG responses in these materials.

## 2. Results and Discussion

### 2.1. Structural Optimization

**Figure 1:**
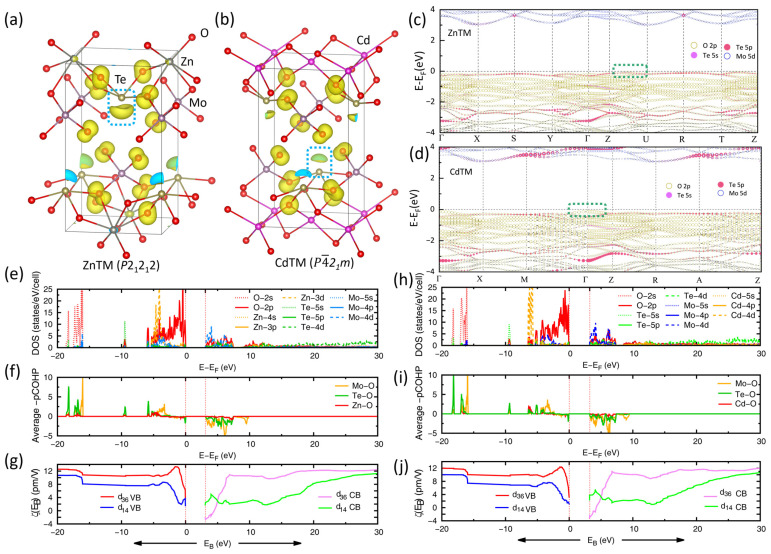
(**a**,**b**) PCD at the valence band maximum (VBM) with an isosurface value of 0.007 for (**a**) ZnTM and (**b**) CdTM, illustrating their different structures. (**c**,**d**) Fat band structures for (**c**) ZnTM and (**d**) CdTM. (**e**–**g**) Electronic structure analysis for ZnTM: (**e**) PDOS, (**f**) -pCOHP (projected crystal orbital Hamilton population), and (**g**) PRF $\zeta_{V}(E_{B})$ and $\zeta_{C}(E_{B})$. (**h**–**j**) Corresponding analysis for CdTM: (**h**) PDOS, (**i**) -pCOHP, and (**j**) PRF $\zeta_{V}(E_{B})$ and $\zeta_{C}(E_{B})$. E_B_ denotes the band energy. Note that the “-” prefix in -pCOHP indicates sign-inverted values, where bonding contributions are plotted as positive.

The relaxed structures of molybdenum tellurite materials MgTM, CdTM, and ZnTM, are shown in Figure 1a,b and Figure S1a. The optimized crystallographic parameters are summarized in Tables S1–S3. A comparison between the calculated structural parameters and the corresponding experimental data shows good agreement, indicating that the optimized structures reliably reproduce the experimental crystal structures. MgTM and ZnTM exhibit similar layered crystal structure with the same orthorhombic space group of *P*2_1_2_1_2. Each unit cell contains three distinct coordination polyhedral in which TeO_4_ tetrahedra formed by Te coordinated with O1 and O2 (TeO1_2_O2_2_), MoO_4_ tetrahedra formed by Mo coordinated with O2 and O3 (MoO2_2_O3_2_), and MgO_6_/ZnO_6_ octahedra formed by Mg/Zn coordinated with O1 and O2 (MgO1_4_O2_2_/ZnO1_4_O2_2_). In contrast, CdTM crystallizes in a different layered structure with a tetrahedra space group of $P\bar{4}$2_1_*m*, as shown in Figure 1b. Each unit cell likewise contains three distinct structural groups, TeO_4_ tetrahedra formed by Te atoms coordinated with O1 and O2 atoms, MoO_4_ tetrahedra formed by Mo atoms coordinated with O2 and O3 atoms, and CdO_4_ tetrahedral groups formed by Cd atoms coordinated with O1 atoms.

It is important to note that the topology of the A-site metal atom layer in CdTM differs significantly from that in ZnTM and MgTM. In orthorhombic CdTM, the Cd atoms on the (001) plane form a centered rectangular (or face-centered square) lattice (Figure S2), whereas Zn and Mg atoms in their respective compounds adopt a simple square lattice. This structural distinction directly results in a much shorter Cd–Cd distance (~3.78 Å) compared to the Zn–Zn distance (~5.31 Å), which induces the change of atom coordination environment and the related chemical interactions. The Mo–O bond lengths remain highly consistent across all three compounds, with Mo–O2 ranging from 1.873 to 1.885 Å and Mo–O3 from 1.722 to 1.725 Å. In contrast, the Te–O bonds show notable variation. While Te–O1 stays nearly constant (1.915–1.935 Å), Te–O2 elongates significantly in CdTM (2.169 Å) and contracts in ZnTM (2.111 Å), indicating greater distortion of the TeO_4_ tetrahedron in CdTM (Table S4). Therefore, although all three compounds share the same stoichiometry, CdTM exhibits distinct crystal structure and coordination environments around the A-site metal compared to the other two compounds.

### 2.2. Electronic Structures

Based on GGA+U calculations performed using VASP, the indirect band gaps (*E*_g_^PBE^) of MgTM, CdTM, and ZnTM are 3.12, 3.22, and 3.10 eV, respectively (Table 1), which are relatively smaller than the corresponding experimental values ($E_{g}^{EXP}$) of 3.12, 3.59, and 3.54 eV. In addition, hybrid functional HSE06 calculations were carried out, yielding band gaps (*E*_g_^HSE^) of 4.39, 4.51, and 4.33 eV for MgTM, CdTM, and ZnTM, respectively, all of which are larger than the experimental band gaps. Therefore, both the experimentally measured band gap (*E*_g_^EXP^) and the HSE06 band gap (*E*_g_^HSE^) were used as lower and upper bounds in the scissor operation (*S*^EXP^ and *S*^HSE^) for the optical property calculations to improve the result reliability. At all computational levels examined, CdTM consistently exhibits the largest band gap, in agreement with experimental observations.

To elucidate the electronic states involved in the optical excitations responsible for the SHG response, we examined the electronic band structures, PDOS, and COHP of the three molybdenum tellurite materials (Figure 1 and Figure S1). In all three compounds, the valence bands (VBs) near the Fermi level (from ~−2.3 eV to E_F_) are predominantly composed of nonbonding O 2p states. Near the VBM, these states exhibit significant Te–O antibonding character (~−1.1 eV to E_F_), which reflects the presence of SCALP electrons on Te atoms, as supported by the -pCOHP analysis (Figure 1f,i and Figure S1d). This conclusion is further corroborated by the partial charge density (PCD) for the VBM and the top VB states (Figure 1a,b and Figure S1a), as well as by the electron localization function (ELF) profiles (Figure S3). Both analyses reveal a highly asymmetric electron density lobe localized on the Te atom along the c axis, confirming the existence of SCALPs in ZnTM and MgTM. However, a distinct contrast is observed in CdTM as its PCD at the k-point of the VBM shows essentially no such localized electron cloud on Te (Figure S4b), indicating a markedly weaker contribution from Te SCALPs (Figure 1d). The diminished NLO accumulation at VBM contribution in CdTM likely originates from a comparatively weaker Te–O interaction. This is evidenced by CdTM having the lowest integrated COHP (-ICOHP) value (4.53 eV, Table S4) among the series, which correlates with a reduced Te–O antibonding interaction near E_F_. This electronic structure difference is also reflected in the fat band representations (Figure 1c,d and Figure S1b). In CdTM, the Te-derived states are primarily concentrated along F–K of the Brillouin zone (BZ). In contrast, for ZnTM and MgTM, the Te contributions are distributed more uniformly across the entire BZ.

Below this region, between approximately −5.8 eV and −2.3 eV, strong Mo–O and Te–O covalent bonding interactions are observed. The conduction bands (CBs), extending approximately from *E*_g_ to 7.5 eV, are mainly composed of O 2p and Mo 4d states, with a smaller contribution from Te 5p orbitals. These CB features correspond predominantly to Mo–O and Te–O antibonding states, with the Mo 4d contribution being notably larger than that of Te 5p. This suggests that the key electronic transitions involved in the optical excitations originate mainly from O–Mo interactions. The principal distinction among the three compounds lies in the position and role of the A-site metal orbitals and their interaction with oxygen. In MgTM, Mg states are negligible near the top of the VBs, consistent with a more ionic character in the Mg–O bonding. In contrast, strong Cd 4d and Zn 3d states appear around −6.0 eV and −4.5 eV, respectively, indicating pronounced covalent bonding character in the Cd–O and Zn–O interactions. We also found that the Cd–O interaction exhibits the highest -ICOHP value (0.95 eV) among the three compounds, indicating the strongest A–O bonding. This is likely influenced by the comparatively narrow Cd–Cd distance within the (001) plane (Figure S2). These differences in the A-site electronic structure are reflected in the variations of bond lengths, polyhedral distortion, and ultimately in the linear and nonlinear optical properties of the three tellurites.

### 2.3. SHG Responses and ART Analysis

The point groups of MgTM and ZnTM are 222, whereas CdTM crystallizes in the $\bar{4}2m$ point group. Consequently, three nonzero SHG tensor components, i.e., *d*_14_, *d*_25_, and *d*_36_, are obtained, as listed in Table 1. In all three compounds, Kleinman symmetry (*d*_14_ = *d*_25_ = *d*_36_) is not strictly obeyed. For this reason, Kleinman symmetry constraints were not enforced in the calculation of nonlinear optical properties throughout this study. Based on the experimental band gaps (*E*_g_^EXP^), the effective SHG coefficients $d_{eff}^{p}$ (averaged over all possible crystal orientations according to the Kurtz–Perry powder method) of MgTM, CdTM, and ZnTM were calculated to be 13.49, 10.34, and 10.86 pm·V^−1^ (Table 1). For cross-validation, SHG coefficients were further evaluated using the ABINIT code with the same experimental band gaps, yielding values of 12.05, 9.38, and 10.03 pm·V^−1^. The results indicate that the SHG responses obtained using the sum-over-states (SOS) method are of the same order of magnitude as those reported by Jiang et al. [24]. When the HSE-calculated band gaps are employed, the corresponding values become 6.76, 6.35, and 7.09 pm·V^−1^ for MgTM, CdTM, and ZnTM, respectively, which align well in magnitude with the results reported by Cammarata et al. [25]. Overall, the computed SHG coefficients show reasonable agreement with available experimental data, though variations exist among different studies (Table 1). These discrepancies can be attributed to differences in computational methods, band gap correction schemes, and experimental measurement conditions.

To quantitatively clarify the origin of the SHG responses in the ATMs, we employed ART combined with the PRF method. By analyzing the energy-dependent PRFs for *d*_36_ and *d*_14_ components, the dominant atomic orbitals and structural units responsible for the SHG can be identified (Figure 1g,j and Figure S1e). For all three ATMs, the SHG responses show a highly consistent microscopic origin. Taking the *d*_36_ component of CdTM as an example, the function (Figure 1j) increases significantly as E_B_ decreases from E_F_ to −1.5 eV, indicating that the occupied nonbonding O 2p states near the VBM contribute strongly to the SHG response. In contrast, it decreases noticeably between −2.3 eV and −5.8 eV, where Mo–O and Te–O bonding interactions dominate, suggesting that these bonding states provide a negative contribution to the SHG response. Meanwhile, the sharp variation of from the CBM up to ~7.5 eV reflects the essential role of unoccupied Mo 4d and Te 5p antibonding states. Similar PRF profiles are observed for the *d*_36_ component in the other two compounds, confirming a general similar electronic-response pattern across the series. Notably, in all three systems, PRF varies only weakly in energy regions associated with A-site cation orbitals, indicating that the direct contributions of these cations to the SHG response are limited. Instead, A-site substitution primarily modifies the crystal field and polyhedral distortion, thereby indirectly tuning the SHG response intensity. Therefore, the SHG responses in these ATMs are determined predominantly by occupied nonbonding O 2p states and unoccupied Mo 4d and Te 5p states, with the A-site cations acting only as secondary tuning factors.

Based on ART, the atomic-scale origin of the dominant SHG coefficient in the ATMs can be quantitatively resolved (Table 2 and Table S5). Here, we take CdTM as a representative example for detailed analysis. In CdTM, the Mo atoms exhibit the highest individual atom contribution (*A*_τ_ ~9.8%), followed by the Te atom (~7.2%). The individual atom contributions of O and Cd are about 5.1% and 2.2%, respectively. Examining the orbital contributions, the O contributions are concentrated in the VB and are predominantly of O 2p character (^VB^_p_*A*_τ_ ~2.8%), consistent with the role of nonbonding O 2p states as the initial states in the SHG process. The VB contribution of Mo in CdTM (~1.7%) arises mainly from Mo 4p orbitals, whereas its CB contribution is dominated almost entirely by Mo 4d states (~7.4%), indicating that the unoccupied Mo 4d states play an important role in the SHG process. The atom also contributes notably to the CB, largely from Te 5p orbitals (~4.1%), though its overall contribution remains smaller than that of Mo. The same trend holds for the other two compounds. When weighted by the number of each atom in the unit cell (W_A_), the total fractional contributions (*C*_A_) of Mo, O, Te, and Cd are 19.6%, 61.3%, 14.3%, and 4.4%, showing that the Mo, O, and Te atoms constitute the primary source of the SHG response in CdTM. The A-site metals (Mg, Cd, and Zn) provide only minor direct contributions (*C*_A_ < 6%), indicating that they act mainly as structural modulators, rather than as primary NLO-active centers.

However, the *d*_14_ component in CdTM presents a distinct behavior. Its $\zeta_{V}(E_{B})$ exhibits a steady increase as E_B_ decreases from E_F_ to −3.1 eV. In contrast, a pronounced peak is observed in the top VBs of ZnTM and MgTM, characterized by a sharp rise at the VBM followed by a sudden decrease around 0.69 eV. The PRF analysis indicates that the synergetic interaction between the Te atom SCALPs and the nonbonding O 2p states in orthorhombic ZnTM and MgTM contributes significantly and positively to the SHG response. In CdTM, however, the SCALPs are relatively weak (PCHG, Figure 1b), failing to induce a similarly drastically contribution in the top VBs. As shown in Table S5, the individual atom contribution of Te atom in VBs (^VB^*A*_τ_) is only 1.6%, which is lower than that of ZnTM (2.8%). This may account for the observed overall decrease in SHG performance in CdTM. It should be noted that, despite the different PRF features of the *d*_14_ component at the top VBs (caused by Te SCALPs in CdTM vs. ZnTM/MgTM), the overall dominant contributors to SHG remain consistent: the occupied nonbonding O 2p states and the unoccupied Mo 4d/Te 5p states. This pattern is similar to that observed for the *d*_36_ component.

According to the individual atomic contributions to the SHG response, the contribution of a given atomic group can be obtained by summing the contributions from the central atom and its coordinated ligands [35]. For example, the group contribution of the MoO_4_ (denoted as [MoO2_2/3_O3_2/1_]) unit to the SHG response in MgTM and ZnTM is calculated as the total contribution of the Mo atom together with those of the surrounding O atoms belonging to this polyhedron:

(1) $$\begin{array}{c} \chi_{\left[{MoO2}_{2/3}{O3}_{2/1}\right]}^{\left(2\right)}=\chi_{Mo}^{\left(2\right)}+2\times \left(\frac{\chi_{O2}^{\left(2\right)}}{3}\right)+2\times \left(\frac{\chi_{O3}^{\left(2\right)}}{1}\right) \end{array}$$

**Figure 2:**
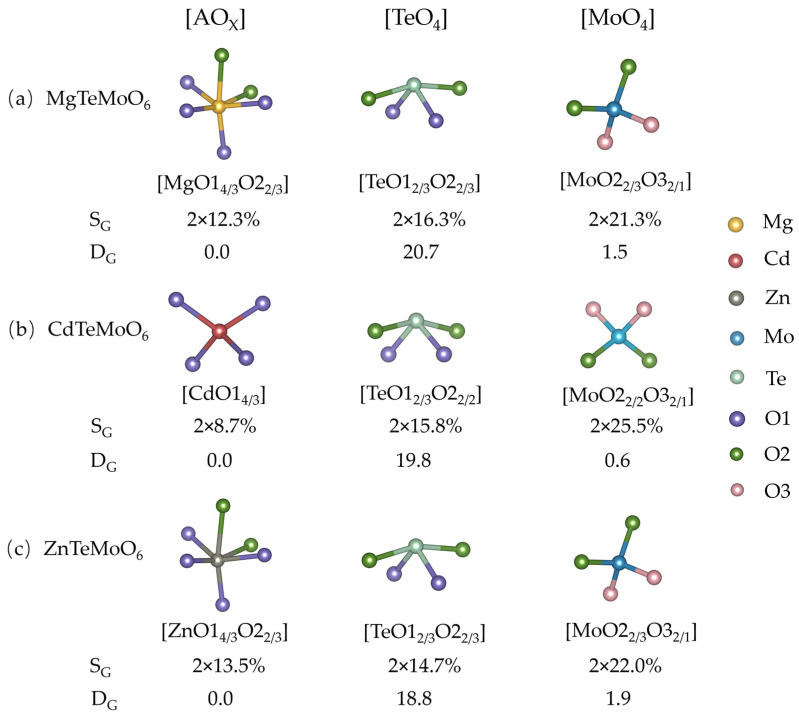
Group contributions to the SHG response (S_G_, in %) and individual group dipole moments (D_G_, in Debye) for (**a**) MgTM, (**b**) CdTM, and (**c**) ZnTM compounds. For A = Mg or Zn, x = 6; for A = Cd, x = 4.

For CdTM, the structural unit is defined as [MoO2_2/2_O3_2/1_] according to its O coordination environment [35]. The total group contribution for the *d*_36_ SHG component for ATMs is presented in Figure 2. In terms of individual group contributions (S_G_), the MoO_4_ groups exhibit the largest values, ranging from 21.3% to 25.5%, which are notably higher than those of the TeO_4_ groups (14.7–16.3%) and the A-centered polyhedra (8.7–13.5%). When considering the total group contribution, the MoO_4_ units account for 42.6–51.0% of the overall response, confirming their dominant role. The TeO_4_ groups also provide a substantial and non-negligible contribution, underscoring their importance in the overall SHG activity of these materials.

We further calculated the dipole moments of individual structural groups (D_G_) for the three compounds and examined their correlation with the corresponding SHG contributions. Note that the three ATMs are intrinsically nonpolar; thus, the net dipole moment of the crystal must vanish. As summarized in Figure 2, the single TeO_4_ groups exhibit the largest dipole moments (18.8–20.7 Debye), followed by the MoO_4_ groups (0.6–1.9 Debye), whereas the metal–oxygen (A–O) groups are nonpolar with zero dipole moments. However, this trend is in sharp contrast to the group SHG contribution analysis, as the group dipole moments and their SHG contributions demonstrate that a large group dipole moment does not necessarily imply a dominant role in generating a strong SHG response. Similar results can be found in our previous analysis on nonpolar KBBF [35]. It was demonstrated that the SHG response does not depend on either the dipole moment of the atomic groups or the static polarization of a crystal.

**Figure 3:**
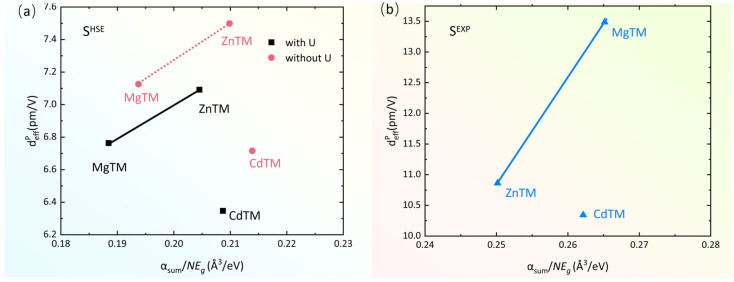
Calculated $d_{eff}^{p}$ and $\alpha_{sum}/(NE_{g})$ for ATMs. (**a**) $d_{eff}^{p}$ values are calculated using scissor S^HSE^ with U (black) and without U (red) correction. (**b**) $d_{eff}^{p}$ values are calculated using scissor S^EXP^ (blue).

In our previous work, we have discovered a linear relationship between $d_{eff}^{p}$ and the $\alpha_{sum}/(NE_{g})$ [36,37,38,39] in several families, where $\alpha_{sum}$ is the sum of the polarizabilities of all ions in the primitive unit cell of a compound [40] (Table S7), N is the total number of atoms in the unit cell, and *E*_g_ is the band gap. However, this linear relationship meets challenges in the three ATM compounds. MgTM and ZnTM share the same crystal structure and a similar feature of PRF. If we use a line to express the relation of $d_{eff}^{p}$ and the $\alpha_{sum}/(NE_{g})$ for MgTM and ZnTM, we naturally get one line (Figure 3). It is clear that the data point for CdTM deviates significantly from this line. This feature holds true regardless of whether the Hubbard U correction is applied or whether *E*_g_^EXP^ or *E*_g_^HSE^ band gaps are used. Given that CdTM crystallizes in a different space group and exhibit a distinct PRF feature for d_14_ component, its deviation from the line formed by MgTM and ZnTM is understandable. Such deviation from one family to the other has been previously noted in other systems such as borates [36] and Pb_2_B_5_O_9_X [38]. Additionally, we found that the direct lines for different structural families often exhibit distinct slopes and intercepts. Such differences may manifest the atomic structure and electronic structure effects. This is certainly a topic worthy of in-depth investigation.

## 3. Methods

The structures and electronic properties of three ATM compounds were calculated using the Vienna ab initio Simulation Package (VASP.5.4.4) [41,42,43] based on density functional theory (DFT). The exchange–correlation interactions were treated using the Perdew–Burke–Ernzerhof (PBE) functional within the generalized gradient approximation (GGA) [44]. The valence electron wave functions were described using the projector augmented-wave (PAW) method [45]. The valence electron configurations of Mg, Cd, Zn, Te, Mo, and O were taken as 2p^6^3s^2^, 4d^10^5s^2^, 3d^10^4s^2^, 5s^2^5p^4^, 4s^2^4p^6^5s^1^4d^5^, and 2s^2^2p^4^, respectively [46]. A plane wave energy cutoff of 600 eV was employed. The Brillouin-zone integrations were carried out with a Γ-centered k-point mesh automatically generated using the KSPACING tag set to 0.12 Å^−1^. Specifically, MgTM and ZnTM compounds were calculated using an 11 × 10 × 6 k-point mesh, while CdTM was calculated with a 10 × 10 × 6 k-point mesh. Structural optimizations were performed until the residual forces on each atom were less than 0.001 eV Å^−1^ and the total energy was converged to within 0.01 meV. Considering that molybdenum tellurite materials contain transition-metal d electrons, the GGA+U approach [47] was employed in our calculations. Based on systematic tests (details provided in Table S8), a U value of 2 eV was applied only to the Mo 4d states.

The second-order NLO properties were calculated using the SOS [48,49,50] method using the ARTAROP.1.6.3 code, which we recently developed [33,34], based on the electronic structures obtained from the VASP optical modules, and SOS methods in ABINIT.9.6.2 code [51,52,53,54]. Since DFT typically underestimates the band gap compared to experimental values, both the experimentally measured band gap (*E*_g_^EXP^) and the HSE06 band gap (*E*_g_^HSE^) were used as lower and upper bounds in the scissor operation (*S*^EXP^ and *S*^HSE^) for the optical property calculations. The SOS formalism for the second-order susceptibility was originally derived by Aversa and Sipe [48] and subsequently modified by Rashkeev et al. [49,50] and Sharma et al. [55,56]. To evaluate the individual atomic and orbital contributions to the SHG tensor components, ART analysis based on the PRF was employed (details provided in Supplementary Materials S1 and S2) [33].

## 4. Conclusions

In summary, based on first-principles calculations combined with the ART, we systematically investigated the electronic structures and the origins of the NLO properties of nonpolar molybdenum tellurite compounds, MgTM, CdTM, and ZnTM. Using quantitative analyses at both the electronic and atomic levels, we reveal that the SHG responses in ATM compounds are mainly governed by the occupied nonbonding O 2p states and the unoccupied Mo 4d and Te 5p states. ART analysis attributes the stronger SHG response in ZnTM and MgTM to an effective and positive synergistic interplay between Te SCALPs and nonbonding O 2p states. However, this mechanism is weakened in CdTM due to diminished Te SCALP activity at VBM, resulting in a lower overall SHG response. From a structural group perspective, the MoO_4_ units contribute approximately 42.6–51.0% of the total SHG response and thus play a dominant role, while the TeO_4_ groups also provide significant and non-negligible contributions. In addition, we find that there is no direct correspondence between the magnitudes of group dipole moments and their SHG contributions. Overall, this work provides a quantitative and microscopic understanding of the physical origins of SHG responses in the ATM family, offering valuable insights into the rational design and development of advanced NLO materials.

## Figures and Tables

**Table 1:** Calculated values of *E*_g_^PBE^, *E*_g_^HSE^, static SHG tensors *d*_ij_, static effective SHG response $d_{eff}^{p}$, and birefringence at 1064 nm compared with available experimental data and literature references.

Compound	MgTM	CdTM	ZnTM
Space group	*P*2_1_2_1_2	$P\bar{4}$2_1_*m*	*P*2_1_2_1_2
*E*_g_^PBE^ (eV)	3.12 ^a^; 3.09 ^b^	3.22 ^a^; 3.17 ^b^	3.10 ^a^; 3.07 ^b^
*E*_g_^HSE^ (eV)	4.39 ^c^	4.51 ^c^	4.33 ^c^
*E*_g_^EXP^ (eV)	3.12 [25]	3.59 [26]	3.54 [27]
*d*_ij_ (pm/V)	*d*_14_ = 15.64 ^a^; *d*_25_ = 16.19 ^a^; *d*_36_ = 16.05 ^a^ *d*_14_ = 13.79 ^b^; *d*_25_ = 13.42 ^b^; *d*_36_ = 15.56 ^b^ *d*_14_ = 7.72 ^c^; *d*_25_ = 7.93 ^c^; *d*_36_ = 8.36 ^c^ *d*_14_ = 14.04 [31]; *d*_14_ = 6.57 [32]	*d*_14_ = *d*_25_ = 12.04 ^a^; *d*_36_ = 12.65 ^a^ *d*_14_ = *d*_25_ = 10.40 ^b^; *d*_36_ = 12.50 ^b^ *d*_14_ = *d*_25_ = 7.27 ^c^; *d*_36_ = 8.00 ^c^ *d*_14_ = 11.75 [31]; *d*_14_ = 14.19 [32]	*d*_14_ = 12.61 ^a^; *d*_25_ = 12.65 ^a^; *d*_36_ = 13.27 ^a^ *d*_14_ = 11.29 ^b^; *d*_25_ = 11.05 ^b^; *d*_36_ = 13.28 ^b^ *d*_14_ = 8.13 ^c^; *d*_25_ = 8.16 ^c^; *d*_36_ = 8.88 ^c^ *d*_14_ = 10.61 [31]; *d*_14_ = 8.64 [32]
$d_{eff}^{p}$ (pm/V)	13.49 ^a^; 12.05 ^b^; 6.76 ^c^; 9.61 [32]	10.34 ^a^; 9.38 ^b^; 6.35 ^c^; 16.96 [32]	10.86 ^a^; 10.03 ^b^; 7.09 ^c^; 12.65 [32]
EXP. SHG (pm/V)	1.5 × KTP [25]; 250 ×α-SiO_2_ [32]	2 × KTP [26,28]; 300 ×α -SiO_2_ [32] *d*_36_ = 8.5 [28];	10.5 × KDP [27]; 250 ×α-SiO_2_ [32]
*Δn* at 1064 nm	0.29 ^a^; 0.26 [31]	0.22 ^a^; 0.16 [31]	0.26 ^a^; 0.23 [31]

^a^ Band gaps are calculated using VASP, and *d*_ij_ and *Δn* are calculated based on the SOS method using VASP + ARTATOP with scissor *S*^EXP^. ^b^ Band gaps are calculated using ABINIT, and *d*_ij_ is calculated based on the SOS method using ABINIT with scissor *S*^EXP^. ^c^ HSE06 band gaps are calculated using VASP, and *d*_ij_ is calculated based on the SOS method using VASP + ARTATOP with scissor *S*^HSE^.

**Table 2:** Contributions of individual atoms to the *d*_36_ SHG component in the ATM compounds. *W*_A_ refers to the number of the same type of atoms (i.e., on the same Wyckoff site) in a unit cell. The total atomic contribution *A*_τ_ represents each atom’s net contribution to the SHG response from both the VBs and CBs, i.e., from all bands. The factor of 1/2 is applied in the definition to avoid double-counting of each excitation process. *C*_A_ represent that from all atoms of the same type. ^VB^*A*_τ_ is the contribution (in %) of the VBs, and ^CB^*A*_τ_ is that from the CBs. The contributions from the *s*, *p*, and *d* states of the atom τ to ^VB^*A*_τ_ and ^CB^*A*_τ_ are also shown. The ART analysis for the *d*_14_ component can be found in Table S5.

Compound	Atom	*W*_A_	*A*_τ_	*C*_A_	^VB^ *A*_τ_	^CB^ *A*_τ_	^VB^_s_ *A*_τ_	^VB^_p_ *A*_τ_	^VB^_d_ *A*_τ_	^CB^_s_ *A*_τ_	^CB^_p_ *A*_τ_	^CB^_d_ *A*_τ_
MgTM	O1	4	6.0	23.9	4.4	1.5	0.5	4.0	0.0	0.3	1.3	0.0
	O2	4	5.1	20.6	3.5	1.7	0.6	2.9	0.0	0.2	1.5	0.0
	O3	4	5.1	20.5	3.6	1.5	1.0	2.6	0.0	0.1	1.4	0.0
	Mg	2	0.9	1.8	0.0	1.0	0.0	−0.1	0.0	0.1	0.4	0.4
	Te	2	8.9	17.7	2.1	6.7	1.0	1.0	0.1	0.4	4.4	1.9
	Mo	2	7.7	15.5	1.2	6.5	0.0	1.3	−0.1	0.2	1.1	5.2
CdTM	O1	4	4.9	19.6	3.4	1.5	0.4	3.0	0.0	0.3	1.2	0.0
	O2	4	5.3	21.2	3.6	1.7	0.6	3.0	0.0	0.1	1.6	0.0
	O3	4	5.2	20.7	3.6	1.6	1.3	2.3	0.0	0.1	1.5	0.0
	Cd	2	2.2	4.5	0.7	1.5	0.0	0.1	0.6	1.5	−0.2	0.3
	Te	2	7.2	14.3	1.4	5.7	0.6	0.7	0.1	0.2	4.1	1.4
	Mo	2	9.8	19.7	1.5	8.3	0.1	1.7	−0.3	0.1	0.9	7.4
ZnTM	O1	4	5.5	22.0	3.9	1.6	0.4	3.5	0.0	0.3	1.3	0.0
	O2	4	5.0	19.9	3.4	1.6	0.5	2.8	0.0	0.2	1.5	0.0
	O3	4	4.8	19.0	3.2	1.6	1.1	2.1	0.0	0.1	1.5	0.0
	Zn	2	2.8	5.6	1.0	1.8	0.0	0.1	0.8	1.4	0.1	0.3
	Te	2	7.7	15.4	1.9	5.8	0.9	0.8	0.1	0.2	4.2	1.4
	Mo	2	9.1	18.1	1.6	7.5	0.0	1.6	−0.1	0.1	0.9	6.4

## Data Availability

The data presented in this study are available in the article and Supplementary Materials.

## Supplementary Materials

The following supporting information can be downloaded at: https://www.mdpi.com/article/10.3390/molecules31050787/s1, S1: Partial response functional (PRF) method; S2: Atom response theory (ART) analysis; Table S1: Optimized crystal structure data for MgTeMoO_6_ (MgTM); Table S2: Optimized crystal structure data for CdTeMoO_6_ (CdTM); Table S3: Optimized crystal structure data for ZnTeMoO_6_ (ZnTM); Table S4: Optimized bond length, distortion index and -ICOHP value of different groups in ATM structures; Table S5: Contributions of individual atoms to the d_14_ SHG component in the ATM compounds. *W*_A_ refers to the number of the same type of atoms (i.e., on the same Wyckoff site) in a unit cell. $A_{\tau }$ is the contribution (in %) from a single atom τ, and *C*_A_ that from all atoms of the same type. ^VB^*A*_τ_ is the contribution (in %) of the VBs, and ^CB^*A*_τ_ from the CBs. The contributions from the *s*, *p*, and *d* states of the atom τ to ^VB^*A*_τ_ and ^CB^*A*_τ_ are also shown; Table S6: Calculated dipole moment of the groups in the ATM compounds; Table S7: Ionic polarizability (α in·Å) and the αsum for each ATM compounds; Table S8: Test of U values (Mo 4d) in three compounds; Figure S1: (a) Partial charge density (PCD) at the valence band maximum (VBM) with isosurface value (0.007), (b) fat band structure, (c) projected density of states (PDOS), (d) crystal orbital Hamilton population (COHP) analysis, and (e) partial response functional (PRF) ζ_V_ (E_B_) and ζ_C_ (E_B_) of MgTM.; Figure S2: Comparison of the A-site metal atom arrangement on the (001) plane in ATM compounds. (a) MgTM and (c) ZnTM adopt a simple square lattice for Mg/Zn atoms; (b) orthorhombic CdTM features a centered-rectangular (face-centered square) lattice formed by Cd atoms; Figure S3: Electron localization function (ELF) plots with isosurface value of 0.85 of ATM compounds on the (001) plane. (a) MgTM, (b) CdTM, and (c) ZnTM; Figure S4: PCD at the k-point of the VBM with same isosurface value (0.007) for (a) MgTM, (b) CdTM, and (c) ZnTM.
