# Supplementary material for: Origin of Large Second-Harmonic Generation in Nonpolar Molybdenum Tellurite Compounds
Source: Molecules. 2026 Feb 26;31(5):787. doi: 10.3390/molecules31050787 (PMC12985411; doi:10.3390/molecules31050787)
Supplement: Supplementary file 1 [file molecules-31-00787-s001.zip › molecules-4133155-supplementary.pdf]

Supporting Information

For

Origin of Large Second-Harmonic Generation in Nonpolar Molybdenum Tellurite Compounds

Zhian Li [1,2,3], Xiyue Cheng [1,3,*], Qian Xu [1,3,4], Xiu Wang [1,3,4], Guoliang Liu [1,2,3] and Shuiquan Deng [1,3,*]

[1] State Key Laboratory of Functional Crystals and Devices, Fujian Institute of Research on the Structure of Matter (FJIRSM), Chinese Academy of Sciences (CAS), Fuzhou 350108, China; lizhian@fjirsm.ac.cn (Z.L.); xuqian@fjirsm.ac.cn (Q.X.); wangxiu@fjirsm.ac.cn (X.W.); liuguoliang@fjirsm.ac.cn (G.L.)

[2] College of Chemistry and Materials Science, Fujian Normal University, Fuzhou 350117, China

[3] Fujian College, University of Chinese Academy of Sciences, Beijing 100049, China

[4] College of Physics and Energy, Fujian Normal University, Fuzhou 350117, China

* Correspondence: xycheng@fjirsm.ac.cn (X.C.); sdeng@fjirsm.ac.cn (S.D.)

S1. Partial response functional (PRF) method [1, 2]

The contribution of a certain occupied energy region between E_B and valence band maximum (VBM), $\zeta_V(E_B)$, to each SHG coefficient $\chi_{ijk}^{(2)}$ is determined by considering only those excitations from all occupied states between E_B and VBM to all the unoccupied states of the conduction bands (CBs), and the contribution, $\delta\zeta_V(E_B)$, of specific occupied states of energy E_B to each $\chi_{ijk}^{(2)}$ by the excitations from that energy to all unoccupied states of the CBs.

$$\delta\zeta_V(E_B) = -\frac{d\zeta_V(E_B)}{dE_B} \tag{1}$$

Similarly, the contribution, $\zeta_C(E_B)$, of a certain unoccupied region between conduction band minimum (CBM) and E_B to each $\chi_{ijk}^{(2)}$ is determined by the excitations from all occupied states of the VBs only to all unoccupied states between CBM and E_B, and the contribution, $\delta\zeta_C(E_B)$, of specific unoccupied states of energy E_B to each $\chi_{ijk}^{(2)}$ by the excitations from all occupied states of the VBs only to that energy.

$$\delta\zeta_C(E_B) = \frac{d\zeta_C(E_B)}{dE_B} \tag{2}$$

S2. Atom response theory (ART) analysis

To evaluate the individual atom contributions to the SHG components, d_{ij}, it is computationally more convenient to express the corresponding PRFs in terms of the band index I_B, $\zeta(I_B)$ [1],where the band index I_B runs from 1 to N_{tot} (i.e., the total number of band orbitals) with increasing energy, E_B, from E_{min} to E_{max}. Here, $\zeta_V(I_B)$ and $\zeta_C(I_B)$ are denoted as ${}^{VB}\zeta_j$ and ${}^{CB}\zeta_j$, respectively, with I_B replaced by a subscript j.

Suppose that a specific atom τ has L atomic orbitals with a coefficient ${}^{VB}C_{L\tau}^{\vec{k}j}$ in the valence band j at a wave vector $\vec{k}$. The total contribution ${}^{VB}A_\tau$ of an atom τ makes to the SHG coefficient from all the VB bands j is written as

$${}^{VB}A_\tau = \frac{\Omega}{(2\pi)^3}\int d\vec{k} \cdot \sum_{L,j} {}^{VB}\zeta_j \left|{}^{VB}C_{L\tau}^{\vec{k}j}\right|^2 \tag{3}$$

where Ω is the unit cell volume, ${}^{VB}\zeta_j$ is the corresponding PRFs in terms of the band index j. Similarly, the total contribution ${}^{CB}A_\tau$ of an atom τ makes to the SHG coefficient from all the CB bands j is written as

$$ {}^{CB}A_\tau = \frac{\Omega}{(2\pi)^3} \int d\vec{k} \cdot \sum_{L,j} {}^{CB}\zeta_j \left| {}^{CB}C_{L\tau}^{\vec{k}j} \right|^2 \tag{4} $$

in which we assumed that the atom has L atomic orbitals with coefficient ${}^{CB}C_{L\tau}^{\vec{k}j}$ in the conduction band j at a wave vector $\vec{k}$. To calculate the actual contribution of each constituent atom in a unit cell to the total SHG response, one needs to consider the signs of ${}^{VB}\zeta_j$ and ${}^{CB}\zeta_j$.

The total contribution, A_τ, each individual atom makes to the SHG response from both the VBs and the CBs (i.e., from all the bands) is given by

$$ A_\tau = \frac{\left({}^{VB}A_\tau + {}^{CB}A_\tau\right)}{2} \tag{5} $$

where the factor of 1/2 is applied to remove the double counting of each excitation.

Table S1. Optimized crystal structure data for $MgTeMoO_6$ (MgTM).

Lattice parameters					
Experiment [3]	a=5.038Å	b=5.269Å	c=8.899Å	α=γ=β=90.00°	Space group
Relaxed	a=5.096Å	b=5.354Å	c=9.143Å	α=γ=β=90.00°	$P2_12_12$
Atoms	x	y	z		Wyckoff sites
Mg	0.000	0.500	0.291		2b
Te	0.000	0.000	0.240		2a
Mo	0.000	0.000	0.695		2a
O1	0.217	0.175	0.103		4c
O2	0.245	0.682	0.203		4c
O3	0.336	0.296	0.419		4c

Table S2. Optimized crystal structure data for $CdTeMoO_6$ (CdTM).

Lattice parameters					
Experiment [4]	a=5.286Å	b=5.286Å	c=9.066Å	α=γ=β=90.00°	Space group
Relaxed	a=5.349Å	b=5.349Å	c=9.382Å	α=γ=β=90.00°	$P\bar{4}2_1m$
Atoms	x	y	z		Wyckoff sites
Cd	0.000	0.000	0.000		2a
Te	0.000	0.500	0.255		2c
Mo	0.000	0.500	0.688		2c
O1	0.192	0.692	0.122		4e
O2	0.218	0.718	0.786		4e
O3	0.681	0.183	0.423		4e

Table S3. Optimized crystal structure data for $ZnTeMoO_6$ (ZnTM).

Lattice parameters					
Experiment [5]	a=5.044Å	b=5.264Å	c=8.919Å	α=γ=β=90.00°	Space group
Relaxed	a=5.117Å	b=5.308Å	c=9.212Å	α=γ=β=90.00°	$P2_12_12$
Atoms	x	y	z		Wyckoff sites
Zn	0.000	0.500	0.018		2b
Te	0.000	0.000	0.241		2a
Mo	0.000	0.000	0.694		2a
O1	0.213	0.182	0.103		4c
O2	0.253	0.692	0.205		4c
O3	0.330	0.298	0.418		4c

Table S4. Optimized bond length, distortion index and -ICOHP value of different groups in ATM structures.

Compound	Groups	Bond (Å)	Bond length (Å)	Distortion index (Å)	$-ICOHP_{av}$ (eV)
MgTM	$MoO2_2O3_2$	Mo-O2	1.877	0.043	7.66
		Mo-O3	1.722		
	$TeO1_2O2_2$	Te-O1	1.917	0.054	4.61
		Te-O2	2.136		
	$MgO1_4O2_2$	$Mg-O1_{av}$	2.171	0.022	0.88
		Mg-O2	2.242		
CdTM	$MoO2_2O3_2$	Mo-O2	1.885	0.044	7.59
		Mo-O3	1.725		
	$TeO1_2O2_2$	Te-O1	1.915	0.062	4.53
		Te-O2	2.169		
	$CdO1_4$	$Cd-O1_{av}$	2.225	0.000	0.95
ZnTM	$MoO2_2O3_2$	$Mo-O2_{av}$	1.873	0.041	7.65
		Mo-O3	1.724		
	$TeO1_2O2_2$	Te-O1	1.935	0.044	4.58
		Te-O2	2.111		
	$ZnO1_4O2_2$	$Zn-O1_{av}$	2.081	0.054	0.87
		Zn-O2	2.385		

Table S5. Contributions of individual atoms to the d_{14} SHG component in the ATM compounds. W_A refers to the number of the same type of atoms (i. e. on the same Wyckoff site) in a unit cell. A_τ is the contribution (in %) from a single atom τ, and C_A that from all atoms of the same type. $^{VB}A_\tau$ is the contribution (in %) of the VBs, and $^{CB}A_\tau$ from the CBs. The contributions from the *s*, *p*, and *d* states of the atom τ to $^{VB}A_\tau$ and $^{CB}A_\tau$ are also shown.

Compound	Atom	W_A	A_τ	C_A	$^{VB}A_\tau$	$^{CB}A_\tau$	$^{VB}{}_sA_\tau$	$^{VB}{}_pA_\tau$	$^{VB}{}_dA_\tau$	$^{CB}{}_sA_\tau$	$^{CB}{}_pA_\tau$	$^{CB}{}_dA_\tau$
MgTM	O	12	4.5	54.0	4.0	0.5	1.2	2.7	0.0	0.0	0.5	0.0
	Mg	2	2.4	4.8	0.1	2.2	0.0	0.0	0.0	0.3	0.7	1.2
	Te	2	5.9	11.8	2.4	3.5	1.5	0.8	0.2	-0.1	-0.2	3.8
	Mo	2	14.7	29.4	7.7	7.0	0.4	5.6	1.6	0.4	0.6	6.0
CdTM	O	12	4.3	51.5	3.9	0.4	1.5	2.5	0.0	0.1	0.3	0.0
	Cd	2	2.6	5.3	1.8	0.9	0.1	0.2	1.5	-0.3	0.1	1.1
	Te	2	5.7	11.5	1.6	4.2	1.0	0.4	0.1	-0.1	-0.1	4.4
	Mo	2	15.9	31.8	9.3	6.6	0.4	7.3	1.6	0.4	0.6	5.5
ZnTM	O	12	4.4	53.4	4.0	0.4	1.3	2.7	0.0	0.0	0.4	0.0
	Zn	2	1.6	3.1	0.5	1.1	0.1	0.2	0.1	-0.4	0.6	0.9
	Te	2	6.8	13.5	2.8	4.0	1.4	1.2	0.2	-0.1	-0.1	4.2
	Mo	2	15.0	30.0	8.6	6.4	0.4	6.3	1.9	0.5	0.7	5.2

Table S6. Calculated dipole moment of the groups in the ATM compounds.

Compound	groups	x	y	z	Magnitude (Debye)
MgTM	MgO_6-1	0.00	0.00	0.00	0.00
	MgO_6-2	0.00	0.00	0.00	0.00
	ΣMgO_6	0.00	0.00	0.00	0.00
	TeO_4-1	0.00	0.00	20.73	20.73
	TeO_4-2	0.00	0.00	-20.73	-20.73
	ΣTeO_4	0.00	0.00	0.00	0.00
	MoO_4-1	0.00	0.00	1.46	1.46
	MoO_4-2	0.00	0.00	-1.46	-1.46
	ΣMoO_4	0.00	0.00	0.00	0.00
	Total	0.00	0.00	0.00	0.00
CdTM	CdO_6-1	0.00	0.00	0.00	0.00
	CdO_6-2	0.00	0.00	0.00	0.00
	ΣCdO_6	0.00	0.00	0.00	0.00
	TeO_4-1	0.00	0.00	19.78	19.78
	TeO_4-2	0.00	0.00	-19.78	-19.78
	ΣTeO_4	0.00	0.00	0.00	0.00
	MoO_4-1	0.00	0.00	0.62	0.62
	MoO_4-2	0.00	0.00	-0.62	-0.62
	ΣMoO_4	0.00	0.00	0.00	0.00
	Total	0.00	0.00	0.00	0.00
ZnTM	ZnO_6-1	0.00	0.00	0.00	0.00
	ZnO_6-2	0.00	0.00	0.00	0.00
	ΣMgO_6	0.00	0.00	0.00	0.00
	TeO_4-1	0.00	0.00	18.77	18.77
	TeO_4-2	0.00	0.00	-18.77	-18.77
	ΣTeO_4	0.00	0.00	0.00	0.00
	MoO_4-1	0.00	0.00	1.85	1.85
	MoO_4-2	0.00	0.00	-1.85	-1.85
	ΣMoO_4	0.00	0.00	0.00	0.00
	Total	0.00	0.00	0.00	0.00

Table S7. Ionic polarizability (α in·Å) [6] and the α_{sum} for each ATM compounds.

Compound	α_A	α_{Te}	α_{Mo}	α_O	α_{sum}
MgTM	0.651	4.430	4.560	1.750	14.891
CdTM	2.700	4.430	4.560	1.750	16.940
ZnTM	1.700	4.430	4.560	1.750	15.940

Table S8. Test of U values (Mo 4d) in three compounds.

Compound	U value	E_g^{PBE} (eV)	E_g^{EXP}(eV)
MgTM	+0	3.04	3.12 [3]
	+1	3.08	
	+2	3.12	
	+3	3.05	
CdTM	+0	3.14	3.59 [4]
	+1	3.15	
	+2	3.22	
	+3	3.15	
ZnTM	+0	3.01	3.54 [5]
	+1	3.05	
	+2	3.10	
	+3	3.14	

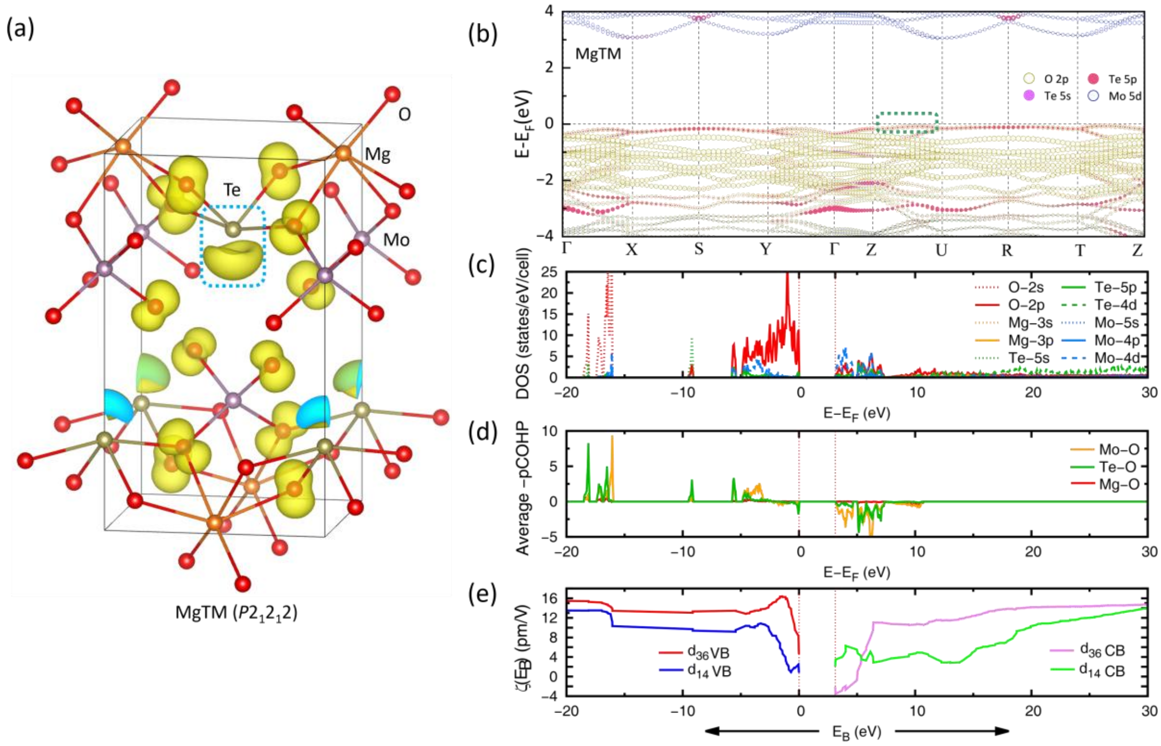

Figure S1. (a) Partial charge density (PCD) at the valence band maximum (VBM) with isosurface value (0.007),(b) fat band structure, (c) projected density of states (PDOS), (d) crystal orbital Hamilton population (COHP) analysis, and (e) partial response functional (PRF) $\zeta_V(E_B)$ and $\zeta_C(E_B)$ of MgTM.

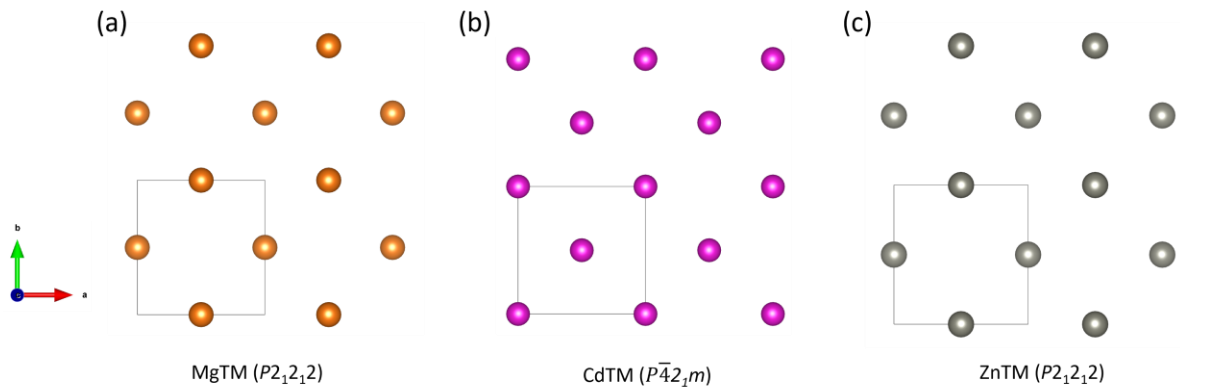

Figure S2. Comparison of the A-site metal atom arrangement on the (001) plane in ATM compounds. (a) MgTM and (c) ZnTM adopt a simple square lattice for Mg/Zn atoms; (b) orthorhombic CdTM features a centered-rectangular (face-centered square) lattice formed by Cd atoms.

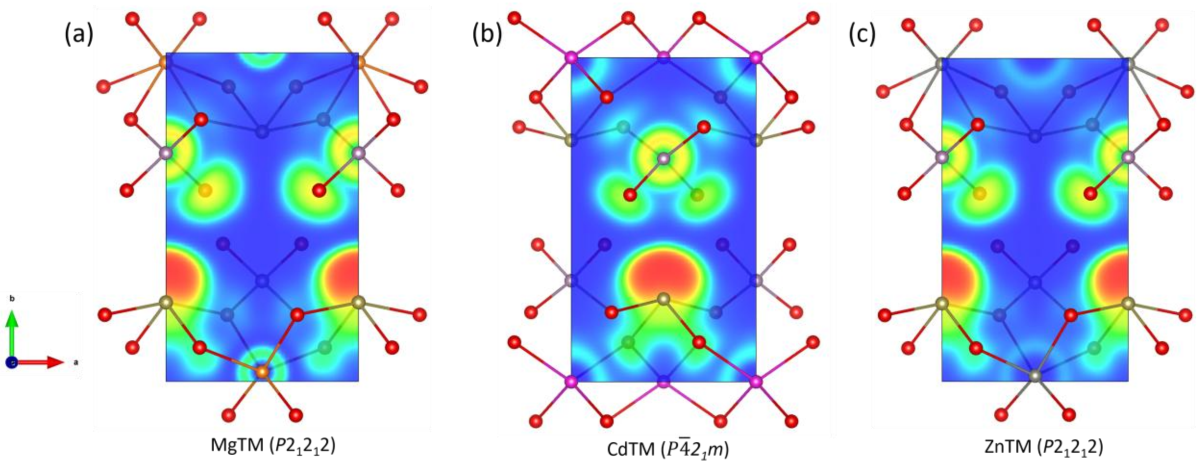

Figure S3. Electron localization function (ELF) plots with isosurface value of 0.85 of ATM compounds on the (001) plane. (a) MgTM, (b) CdTM, and (c) ZnTM.

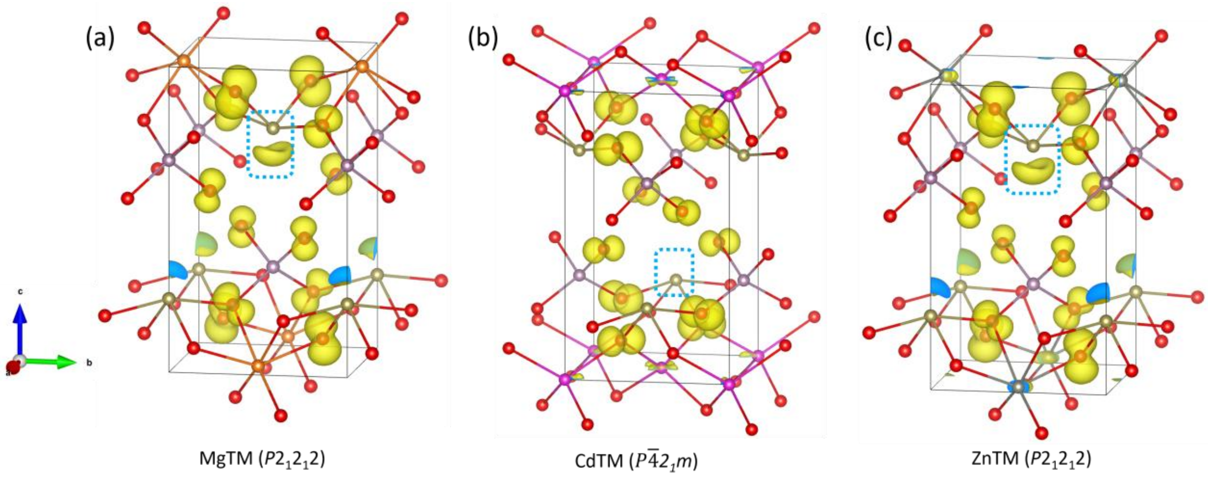

Figure S4. PCD at the k-point of the VBM with same isosurface value (0.007) for (a) MgTM, (b) CdTM, and (c) ZnTM.

References

1. Cheng, X.; Whangbo, M.H.; Guo, G.C.; Hong, M.; Deng, S., The large second-harmonic generation of $LiCs_2PO_4$ is caused by the metal-cation-centered groups. *Angew. Chem. Int. Ed.* **2018,** 57, (15), 3933-3937.
2. Cheng, X.; Whangbo, M.H.; Hong, M.; Deng, S., Dependence of the second-harmonic generation response on the cell volume to band-gap ratio. *Inorg. Chem.* **2019,** 58, (15), 9572-9575.
3. Zhang, J.; Zhang, Z.; Sun, Y.; Zhang, C.; Zhang, S.; Liu, Y.; Tao, X., $MgTeMoO_6$: A neutral layered material showing strong second-harmonic generation. *J. Mater. Chem.* **2012,** 22, (19), 9921-9927.
4. Zhao, S.G.; Jiang, X.X.; He, R.; Zhang, S.Q.; Sun, Z.H.; Luo, J.H.; Lin, Z.S.; Hong, M.C., A combination of multiple chromophores enhances second-harmonic generation in a nonpolar noncentrosymmetric oxide: $CdTeMoO_6$. *J. Mater. Chem. C* **2013,** 1, (16), 2906-2912.
5. Zhao, S.G.; Luo, J.H.; Zhou, P.; Zhang, S.Q.; Sun, Z.H.; Hong, M.C., $ZnTeMoO_6$: A strong second-harmonic generation material originating from three types of asymmetric building units. *RSC Adv.* **2013,** 3, (33), 14000-14006.
6. Shannon, R.D.; Fischer, R.X., Empirical electronic polarizabilities of ions for the prediction and interpretation of refractive indices: Oxides and oxysalts. *Am. Mineral.* **2016,** 101, 2288-2300.
